# A comparison of the clinical features of molar pregnancy in adolescents and adults

**DOI:** 10.12669/pjms.40.5.8383

**Published:** 2024

**Authors:** Mehmet Ozer, Pınar Tugce Ozer, Ibrahim Karaca, Suna Karaca, Alper Ileri, Adnan Budak

**Affiliations:** 1 Mehmet Ozer, Dept. of Obstetrics & Gynaecology, University of Health Sciences, Tepecik Training and Research Hospital, Izmir, Turkey; 2 Pınar Tugçe Ozer Dept. of Obstetrics & Gynecology, Izmir Economy University, School of Medicine, Medical Point Hospital, Izmir, Turkey; 3 Ibrahim Karaca Dept. of Obstetrics & Gynaecology, Izmir Bakircay University, Cigli Education and Research Hospital, Izmir, Turkey; 4 Suna Karaca, Dept. of Obstetrics & Gynaecology, University of Health Sciences, Tepecik Training and Research Hospital, Izmir, Turkey; 5 Alper İleri, Dept. of Obstetrics & Gynaecology, University of Health Sciences, Tepecik Training and Research Hospital, Izmir, Turkey; 6 Adnan Budak, Dept. of Obstetrics & Gynaecology, University of Health Sciences, Tepecik Training and Research Hospital, Izmir, Turkey

**Keywords:** Gestational trophoblastic diseases, Hydatidiform mole, Vaginal bleeding, Visual analogue scores

## Abstract

**Objective:**

To compare the age-specific clinical features of molar pregnancy and to describe the risk factors associated with this situation.

**Method:**

This retrospective case-control study was conducted at the Department of Obstetrics and Gynecology. Tepecik Education and Research Hospital, Izmir, Turkey. The participants included both adolescents (≤ 19 years) and adults with histologically confirmed hydatidiform moles in our institution between January 2015 and January 2022. The interventions and main outcome measures of this study involved evaluating the clinical and ultrasonographic features, as well as the risk factors, associated with molar pregnancies in adolescents.

**Results:**

This study of 137 patients with molar pregnancy found that adults had a higher incidence of partial molar pregnancy (20 patients versus seven patients) and lower beta-hCG levels than adolescents (176.890.71 mIU/ml versus 253.734.47 mIU/ml). Adolescents had a higher likelihood of hyperthyroidism (25.4% versus 9.2%). bleeding on admission (4.2% versus 1.51%),. longer hospital stays (5.44 ± 2.73 days versus 3.59 ± 3.08 days). Higher rates of uterine enlargement and postoperative bleeding (15.5% versus 1.5%). Adolescents also required more analgesia (97% versus 89.4%).

**Conclusions:**

Adolescents with Gestational trophoblastic diseases (GTD) may present with more severe symptoms compared to adults, which can lead to delayed diagnosis and treatment. Further research is needed to better understand the underlying mechanisms and risk factors for GTDs in this population. Increased awareness and education can help improve recognition and management of GTDs in adolescents and improve their overall health outcomes.

## INTRODUCTION

Gestational trophoblastic diseases (GTD) are pregnancies that originate from the placenta and can be either benign or malignant.^1^ Studies on the incidence of the disease have shown wide geographical variation.^2^ In the United States, the incidence has been reported to be approximately one in 1500 pregnancies, while in Asia. the rate is generally higher, at around one in 500. Another study conducted in Stockholm reported a rate of 2.8 in 1000 births, which is said to increase over time.^2^-^4^

A history of previous molar pregnancies is the most important factor that increases the risk of developing GTD.^4^ In addition to a history of molar pregnancy, another criterion that increases the risk is maternal age being over 45 or under 15 years old.^5^,^6^ In the pathogenesis of molar pregnancies in older mothers, defective oocytes are thought to be selected for fertilization at the beginning and end of the reproductive period. In the adolescent age group, the situation is blamed on different causes. At the start of the reproductive period, ovulation defects may occur due to the immature endocrine system.^5^

Patients may present with symptoms such as vaginal bleeding, pelvic pain, and hyperemesis gravidarum in the first trimester. Abnormally high beta-HCG levels and the presence of theca-lutein cysts larger than 6cm suggest a molar pregnancy.^4^ The “snowstorm” appearance seen on ultrasound also supports the diagnosis.^7^ Often, the diagnosis is made based on pathology results obtained after spontaneous abortion.^5^ In later weeks of pregnancy, symptoms of hyperthyroidism such as sweating and tachycardia may accompany the disease.^7^ In nearly 20% of cases with malignant transformation, brain and lung metastases can be observed after curettage.^4^

Although early diagnosis and treatment are important at any age,^7^ adolescent pregnancies are at higher risk due to being further from physiological norms. GTD in adolescent patients is very rare. Due to the difficulties in treatment and follow-up adherence of patients, mortality, and morbidity can be observed at higher rates. Recognizing and treating this rare condition is important for these patients to have healthy pregnancies in the future.^8^

Due to limited data on gestational trophoblastic diseases in the adolescent age group, it is not known whether the clinical features of the disease are different from those of adults, which can lead to delayed diagnosis. In this study, we aimed to raise awareness of gestational trophoblastic diseases in the adolescent age group, evaluate their outcomes, and improve adolescent health status.

## METHODS

This retrospective case-control study analyzed adolescent girls with histologically confirmed hydatidiform moles at Health Sciences University Izmir Tepecik Training and Research Hospital between January 2015 and January 2022. The hospital serves as a tertiary center for the country’s third-largest city and facilitates over 10,000 births per year. The study aimed to evaluate clinical and ultrasonographic features and risk factors for adolescent molar pregnancies.

### Ethical Approval:

The ethics committee of Izmir Tepecik Training and Research Hospital approved the study with the approval numbered 2022/08-08 dated 15-08-2022. Patient information was collected using the “probell” database of the hospital.

The diagnosis of molar pregnancy was confirmed using the morphologic criteria described by Ghassemzadeh et al.^9^ Patients underwent a clinical and pre-anesthetic evaluation, including various tests such as a complete metabolic profile, complete blood count, chest radiograph, and serum quantitative hCG before uterine aspiration. Patients were found to be in the first trimestr of pregnancy based on the last menstrual period during the diagnosis. Pelvic-transvaginal ultrasonography was performed on all patients, and additional tests such as thyroid-stimulating hormone, free thyroxine, and electrocardiogram were obtained if required to assess thyroid and cardiac function.

All patients underwent electrical vacuum aspiration and the same follow-up procedures. Patients with incomplete medical records or those who discontinued follow-up were excluded. Out of 209 patients diagnosed with molar pregnancy histologically, 78 were adolescents. Only 71 of these patients with accessible medical records were included in the study, and the control group comprised patients over 19 years of age with a diagnosis of molar pregnancy (n=66 patients). Visual Analogue Scores (VAS) of patients were recorded and patients have more than five accepted as positive. The control group selected from patients who were admitted at same dates and matched with adolescent group by using statistical methods. During clinical examination, excessive uterine size was considered when the uterus was at least four weeks larger than expected for gestational age.^10^

After vacuum curettage, the patients underwent weekly hCG control until three negative values (<5 mIU/mL) were obtained. Following this, monthly controls were initiated, and patients were followed up until three negative values were detected in monthly controls. Physical and pelvic examinations were performed every two weeks for three months and then once a month for the remaining nine months. Uterine involution was evaluated at each examination as subinvolution of the uterus and accompanying abnormal uterine bleeding are warning signs of persistent disease. Patients were recommended to use oral contraceptives for 6-12 months for contraception and to differentiate persistent or recurrent disease. They were informed about the importance of regular follow-ups and advised to have all analyses performed in the same laboratory to ensure that they were performed with the same commercial assay and limit uncertainty in variations in hCG levels.

### Statistical analysis:

It was conducted using IBM SPSS Statistics Standard Concurrent User V 26 (IBM Corp., Armonk, New York, USA). Descriptive statistics were reported as number (n), percentage (%), mean ± standard deviation (SD), median (M), 25th percentile (Q1), and 75th percentile (Q3) values. Normality of numerical data was assessed using the Shapiro-Wilk test and Q-Q plots, while homogeneity of variances was assessed using the Levene test. For univariate analysis, independent two-sample t-tests or Mann-Whitney U tests were used for numerical variables, depending on normality. Pearson chi-square test with the exact method was used for comparison of categorical variables. Variables with a significance level of p<0.10 were subjected to binary logistic regression analysis, and the backward elimination Wald method was used to determine the final variables. A p-value of less than 0.05 was considered statistically significant in logistic regression analysis.

## RESULTS

A total of 137 patients were enrolled in this study, with a mean age of 22.89 ± 8.44 years. Upon examination of the ultrasonography images, the mean size of the intrauterine mole was found to be 58.64 ± 26.7 mm. while the mean hemoglobin levels before curettage were 12.25 ± 8.63 g/dl. After curettage, the hemoglobin levels were 10.25 ± 1.10 g/dl. 20 (17%) of our followed-up patients had experienced a recurrent molar pregnancy during follow-up.

In terms of patient demographics, 71 patients (51.8%) were in the adolescent group. Gravidity, parity, live birth, hemoglobin levels before and after curettage, time to clearance of beta-hCG levels, symptoms and intrauterine mole size were compared between the adolescent and adult groups, and the results are presented in Table-I.

**Table-I:** Comparison of clinical characteristics of patients with molar pregnancy.

Clinical Features	Adolescents (≤19 years) (n=71 patients)	Adults (>19 years) (n=66 patients)	p-value
Median Age (min-max)	18 (14-19)	27 (20-48)	0.001
Median Gravidity (min-max)	1.39	2.53	0.001
Median Parity (min-max)	0.39	1.30	0.001
BMI (kg/m2)	24.3±3.2	25.4±3.3	0.35
Asymptomatic. n (%)	22 (34.3%)	72 (59%)	0.01
Vaginal bleeding. n (%)	34 (47.8%)	7 (10.6%)	0.012
Hyperemesis gravidarum. n (%)	20 (31.2%)	26 (21.3%)	0.132
Hyperthyroidism. n (%)	18 (25.4%)	6 (9.2%)	0.012
Hcg>100.000 on presentation. n (%)	45 (70.3%)	66 (54%)	0.032
Hcg>50.000 on presentation. n (%)	56 (87.5%)	82 (67.2%)	0.003
Drop in hemoglobin levels. g/dl	1.76±0.6	1.85±1	0.73
Mole type. n (%) Partial	7 (9.8%)	20 (30.3%)	0.001
Time to Hcg normalization. day	60.32	54.35	0.22
Length of hospital stay. day (mean±SD)	5.44 ± 2.73	3.59 ± 3.08	<0.001
Method of removal. n (%) Dilation and curettage Hysterectomy. n (%)	64 (100%) 0	117 (96%) 5(4%)	0.13NA
Increased uterine volüme. n (%)	42 (62.5%)	70 (57.3%)	0.275
Intrauterine snowstorm appereance. n (%)	35 (49.3)	25 (37.8)	0.008
Theca lutein cysts. n (%)	5 (7%)	9 (7%)	0.82
Intraoperative complications. n (%)	0	1	NA
Postoperative Bleding. n (%)	11 (15.5)	1 (1.5)	0.002
GTN. n (%)	6 (8.45%)	7 (10.6%)	0.08
VAS > 5	69 (97%)	59 (89.4%)	0.004

BMI: Body Mass Index. GTN: Gestational Trophoblastic Neoplasia. HCG: Human chorionic gonadotropin. VAS: Visual Analog Score

Analysis of mole pregnancy types revealed that partial mole pregnancy occurred in seven patients (9.8%) in the adolescent group, while this number was 20 (30.3%) in the adult group, demonstrating a significantly higher incidence in adults (p=0.001).

When examining patients with complete mole pregnancy, the mean beta-hCG value before curettage was 253,734.47 mIU/ml in adolescents and 176,890.71 mIU/ml in adults. This difference was statistically significant (p=0.006).

The length of hospital stay was significantly different between adolescents (5.44 ± 2.73 days) and adults (3.59 ± 3.08 days) (p<0.001). In addition, while the uterus diameter of 50 patients (70.4%) in the adolescent group exceeded normal limits, this number was 48 (48.72%) in the adult group, and the difference was statistically significant (p=0.018). Intrauterine snowstorm appearance was observed in 35 patients (49.3%) in the adolescent group and in 25 patients (37.8%) in the adult group, with a significantly higher likelihood in adolescents (p=0.008).

Furthermore, hyperthyroidism was significantly more common in adolescents (25.4%) than in adults (9.2%) (p=0.012). Bleeding on admission was also observed more frequently in adolescents (34 patients: 47.8%) than in adults (seven patients: 10.6%) (p=0.012). The development of gestational trophoblastic neoplasia (GTN) was not statistically significantly different between adolescents (six patients: 8.45%) and adults (seven patients: 10.6%) (p=0.08). However, the risk of postoperative bleeding was significantly higher in adolescents (11 patients: 15.5%) than in adults (one patient: 1.5%) (p=0.002). Finally. 97% (69) of the adolescent patient group had VAS>5. while this ratio was 89.4% (59) in adults.

## DISCUSSION

The study’s conclusions indicate that there has been a statistically significant increase in the rates of asymptomatic pregnancies among adolescents, vaginal bleeding, hyperthyroidism, length of hospitalization, postoperative bleeding risk, and a VAS score >5. Furthermore, teenagers had a noticeably decreased rate of partial molar pregnancies. However, when it came to the duration of HCG normalization, uterine volume increase, incidence of theca lutein cysts, amount of hemoglobin level decrease. BMI values, incidence of hyperemesis gravidarum, HCG levels at presentation, intraoperative complications, and incidence of gestational trophoblastic neoplasia (GTN), no statistically significant differences were found between the groups. Adolescent pregnancies pose a significant public health issue, both physiologically and socially. Delayed detection of these pregnancies, non-compliance with antenatal care, and inadequate knowledge of risks increase maternal mortality and morbidity.^8^ Early and late peaks of molar pregnancies, especially in the adolescent age group, require attention, Suspecting the disease and making an early diagnosis can reduce mortality and morbidity in patients.

The clinical presentation of GTD has undergone significant changes in recent years due to early diagnosis, which is associated with increased use of ultrasound during pregnancy. Classic symptoms such as vaginal bleeding, increased uterine volume, and the presence of corpus luteum cysts are less frequently observed nowadays. Studies suggests a trend towards a decrease in the frequency of symptoms and signs, along with an increase in the number of asymptomatic patients diagnosed.^11^.^12^

In this study, we observed that GTDs in adolescents are more symptomatic compared to adults. Adolescents presented with a higher incidence of vaginal bleeding and had higher levels of human chorionic gonadotropin (hCG) than adults. These findings suggest that questioning symptoms and physical examination are quite important at adolescents also management of GTD in adolescents could be more difficult. Other studies have also found that vaginal bleeding is seen at an earlier age compared to other symptoms.^8^

In our study, approximately 47.8% of adolescents presented with vaginal bleeding, which was more frequent compared to adults. This was consistent with other studies. Additionally, we identified a higher incidence of complete mole in the adolescent cohort compared to the adult group, revealing a notable association with increased occurrences of vaginal bleeding and elevated B-HCG levels within this demographic. This observation aligns with the findings of a national study^13^ on Gestational Trophoblastic Disease (GTD), where a comprehensive analysis of complete and partial mole cases indicated parallel outcomes to our research. Notably, the complete mole group exhibited a heightened frequency of vaginal bleeding symptoms and elevated B-HCG levels. This consistent pattern underscores the generally symptomatic nature of complete mole cases, with a particular emphasis on vaginal bleeding as a noteworthy and clinically significant symptom. Although vaginal bleeding remains a common symptom, it has significantly decreased compared to the past.Click or tap here to enter text.^14^ In this study, factors such as decreased awareness of pregnancy-related issues among adolescents, and concerns regarding gynecological examination, may contribute to the higher frequency of symptomatic cases in this population compared to older patients. We observed that socioeconomic level of adolescent GTD patients are lower than the adults. These findings have important implications for healthcare providers and highlight the need for improved education and awareness campaigns to promote early diagnosis and effective management of pregnancy-related conditions.

In this study, we thought that prophylactic hysterectomy was a viable option for adults who had completed their fertility requests and later developed gestational trophoblastic disease (GTD). According to Zhao et al.’s review, comparing treatment options of hysterectomy and evacuation in patients over 40 years old, there was a significant reduction in the incidence of postmolar GTD development among those who underwent hysterectomy.^15^ These findings suggest that hysterectomy may be an alternative treatment option for this patient population. However, in the adolescent group, six individuals developed GTN and were treated with chemotherapy.

In this study, the development of complete mole was observed to occur at a higher rate compared to partial mole in adolescents. Additionally, the development of complete mole was found to be more prevalent in the adolescent group compared to the adult group, when compared to the development of partial mole. In a study conducted by Gockley et al. the risk of developing complete mole was found to be seven times higher in adolescents compared to the general population, while the risk of partial mole was similar to that of the general population.^16^

According to the findings from this study, there appears to be a higher likelihood of complete molar pregnancy occurring at the extremes of reproductive age. Factors such as immature reproductive system and immature ovary may contribute to a higher probability of ovulation with an empty ovum. However, the specific mechanism behind the increased occurrence of molar pregnancies in adolescent patients remains largely uncertain. These findings suggest that age may be an important factor in the development of complete mole, and further research is needed to determine the underlying mechanisms contributing to this increased risk in adolescents.

The most important way to prevent molar pregnancies in the adolescent age group is to know about contraception methods. The importance of education on this subject is significant. Although literature studies have reported an association between early diagnosis of complete hydatidiform mole and a decrease in classical symptoms such as bleeding, anemia, and excessive uterine size, our study group showed that more than 50% of the cases exhibited an increased uterine size.^10^

Considering all these factors, molar pregnancies in adolescents should be recognized and treated at an early stage. Additionally, it is known that molar pregnancies can recur at a rate of approximately 1.8%.^5^ Individuals should have regular follow-up after treatment, and their subsequent pregnancies should be monitored. Taking all these measures is significant in reducing maternal morbidity and preventing mortality.

This study makes a substantial contribution to the literature by comparing the clinical characteristics of molar pregnancies in adolescents and adults. A number of variables that have not been previously recognized or highlighted in the literature are revealed by increases in determinant factors, including rates of asymptomatic pregnancies, vaginal bleeding, hyperthyroidism, length of hospitalization, postoperative bleeding risk and VAS score among teenagers. Additionally, the observed decrease in the rate of partial molar pregnancies in adolescents constitutes a significant finding, indicating variations in pregnancy types among age groups. The study brings new insights to the literature on the early diagnosis and successful treatment of patients in this age group by highlighting the tendency for gestational trophoblastic disorders (GTD) in teenagers to have a symptomatic course. The findings highlight the need of healthcare providers creating specialized awareness campaigns and educational plans for this demographic, as molar pregnancies may cause symptoms to worsen during adolescence.

The retrospective design of this study has made it possible to investigate, within more constrained parameters the features of molar pregnancies in adult and adolescent populations. It has also been difficult to demonstrate a causal relationship between the findings and the small number of individuals with unusual molar pregnancies. Future research should focus on using larger sample numbers and prospective designs to get around these restrictions.

## CONCLUSION

This study contributes valuable insights to clinical practice by comparing the clinical features of molar pregnancy in adolescents and adults. It highlights the differences in symptom severity between these two ages groups and underscores the significance of early recognition and management of molar pregnancies in adolescents. By providing evidence of more severe symptoms in adolescents. the study emphasizes the need for heightened awareness and education among healthcare providers to enhance the identification and treatment of molar pregnancies in this population. The findings of this study have the potential to enhance patient care and improve health outcomes for adolescents with gestational trophoblastic diseases.
